# Supplementary material for: Interventions for vulnerable pregnant women: Factors influencing culturally appropriate implementation according to health professionals: A qualitative study
Source: PLoS One. 2022 Aug 3;17(8):e0272249. doi: 10.1371/journal.pone.0272249 (PMC9348690; doi:10.1371/journal.pone.0272249)

**Topic list: Interventions for vulnerable pregnant women: factors influencing implementation according to health professionals: a qualitative study (Dutch)**

De topiclijst die gebruikt werd was gebaseerd op het kwetsbaarheidsmodel van Briscoe et al. , 2016 (figuur 1) en op de checklist determinanten van innovaties in gezondheidsorganisaties van Fleuren et al. 2014 (figuur 2) en 2012 (figuur 3).

1. Achtergrondkarakteristieken (o.a.: praktijk/organisatie: grootte, stad/platteland, VSV; prof: beroep, leeftijd, werkervaring algemeen en met kwetsbare vrouwen in het bijzonder)
2. Wanneer is/noemen jullie een (zwangere) vrouw *kwetsbaar*?
   1. Denk aan kwetsbaarheidsmodel van Briscoe, et al. Zie figuur 1
3. Welke interventies worden aangeboden aan kwetsbare zwangere vrouwen?
4. Met welke implementatiegraad?:
   1. adoptie, implementatie, consolidatie, verspreiding (zie figuur 2). Leg de focus op implementatie (Fleuren, et al., 2014)
5. Wat zijn de ervaren belemmerende en bevorderende factoren voor de implementatie en effectiviteit van interventies voor kwetsbare zwangeren vanuit zowel het perspectief van vrouwen zelf als van professionals in de geboortezorg? Wat kan er worden gedaan om de interventies beter te laten slagen? Waarom lukt het momenteel niet zo goed om interventie te laten slagen?
   1. Denk aan MIDI-determinanten (zie figuur 3, Fleuren, et al, 2012)
      1. bij zwangere
      2. bij je zelf en collega’s als zorgverleners
      3. organisatie
      4. interventie zelf
      5. sociaal-politiek klimaat, politiek, rol van zorgverzekeraars, gemeenten
6. Is er verschil tussen de aangeboden interventies? Te maken met verschillende randvoorwaarden (locatie, begeleiding, werving e.d.)?

Referenties:

- Briscoe L, Lavender T, McGowan L. A concept analysis of women's vulnerability during pregnancy, birth and the postnatal period. J Adv Nurs 2016;72(10):2330-2345.
- Fleuren MA, Paulussen TG, Dommelen P van, Buuren S van. Meetinstrument voor Determinanten van Innovaties (MIDI). ©TNO, 2012.
- Fleuren MAH, Paulussen TGWM, Van Dommelen P, Van Buuren S. Towards a measurement instrument for determinants of innovations. Int J Qual Heal Care. 2014;26(5):501–10.

Bijlagen:

***Figuur 1. Het kwetsbaarheidsmodel van Briscoe et al. 2016 aangepast met Midi-determinanten (*Fleuren, et al, 2012)**


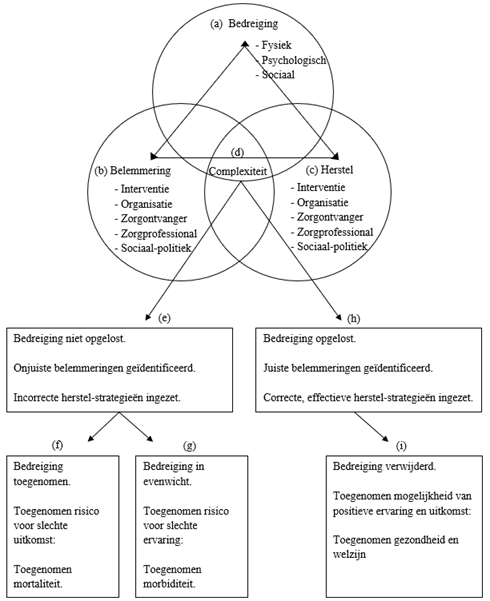


*Figuur 1. Deel van het kwetsbaarheidsmodel van Briscoe et al (2016) met 3 thema’s die met hun aan- of/en afwezigheid de kwetsbaarheid bepalen. Interactie tussen deze 3 thema’s beïnvloedt de mate van kwetsbaarheid. Overlapping van deze 3 thema’s zorgt voor een 4de thema, hier genoemd complexiteit. Onder de 3 thema’s de 5 subthema’s volgens het aangepaste model van Fleuren et al., 2014*

***Figuur 2 Kader: implementatieproces en gerelateerde categorieën en determinanten***


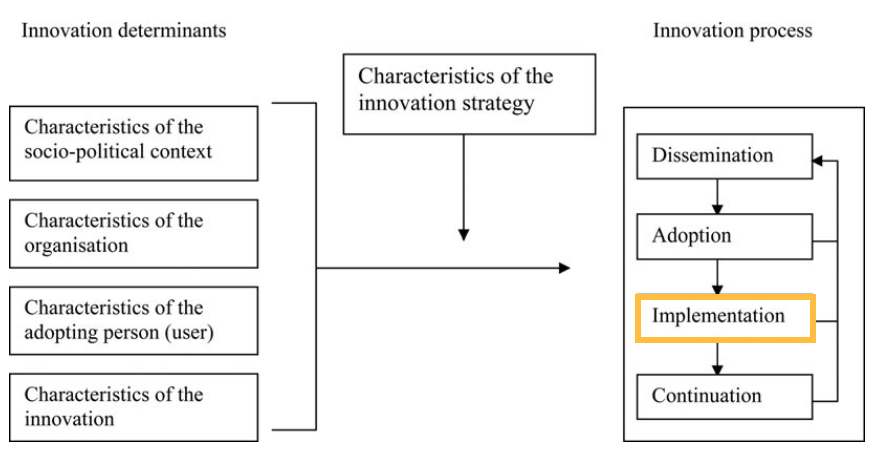


*In de interviews is de focus gelegd op de ‘innovation determinants’, ofwel de factoren van invloed op de implementatie van de interventie. Hierbij is vooral gekeken naar de factoren die betrekking hebben op de derde fase in het ‘innovation process’, de implementatie-fase. Gedurende deze fase wordt de vernieuwing in de dagelijkste routine opgenomen* (Fleuren, et al, 2014).

***Figuur 3 Categorieën en determinanten uit het MIDI (Fleuren, et al., 2012)***


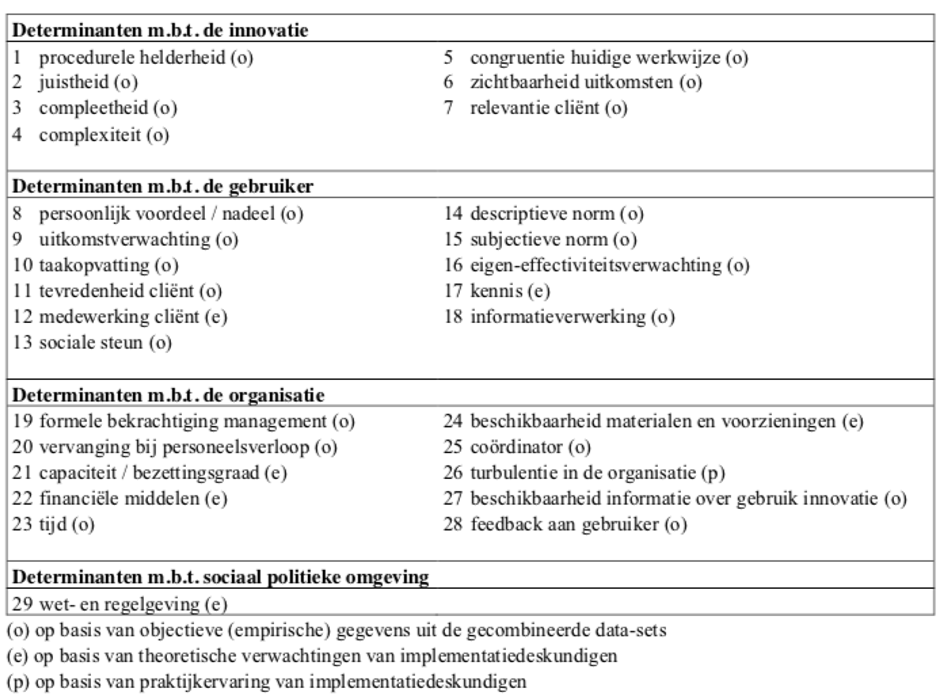

Supplement: S1 File — (DOCX) [file pone.0272249.s001.docx]
