# Supplementary material for: Interventions for vulnerable pregnant women: Factors influencing culturally appropriate implementation according to health professionals: A qualitative study
Source: PLoS One. 2022 Aug 3;17(8):e0272249. doi: 10.1371/journal.pone.0272249 (PMC9348690; doi:10.1371/journal.pone.0272249)
Supplement: S2 File — (DOCX) [file pone.0272249.s002.docx]

**Topic list Interventions for vulnerable pregnant women: factors influencing implementation according to health professionals: a qualitative study**

**Date: 20190115**

The topic list was based on the vulnerability model of Briscoe et al., 2016 (Figure 1) and on the checklist of determinants of innovations in health organizations by Fleuren et al. 2014 (Figure 2) and Fleuren et al. 2012 (Figure 3).

Note for interviewers: the points that are written in italic are reminders for you! You don’t have to ask the participants these concepts.

1. Background characteristics of participants (including:
   1. practice/organization: size, level of urbanization (urban/rural), obstetric cooperation units;
   2. professional: profession, age, work experience in general and with vulnerable women in particular.
2. When is/do you call a (pregnant) woman *vulnerable*?
   1. *See the vulnerability model of Briscoe, et al. See figure 1*
3. What interventions are offered to vulnerable pregnant women?
4. Degree of implementation:
   1. *adoption, implementation, consolidation, dissemination (see figure 2). Focus on implementation (Fleuren, et al., 2014)*
5. What factors are perceived hindering and/or facilitating the implementation and effectiveness of interventions for vulnerable pregnant women from the perspective of both women as the health professionals? What can be done to make the interventions more successful?
   1. *See determinants (see figure 3, Fleuren, et al, 2012) of Measurement Instrument for Determinants of Innovations (MIDI)*
      1. *in pregnant women*
      2. *with yourself and colleagues as health professionals*
      3. *organization*
      4. *intervention itself*
      5. *socio-political climate, politics, role of health insurers, municipalities*
6. Is there a difference between the interventions offered? Do you have to deal with different conditions (location, supervision, recruitment, etc.)?

References:

- Briscoe L, Lavender T, McGowan L. A concept analysis of women's vulnerability during pregnancy, birth and the postnatal period. J Adv Nurs 2016;72(10):2330-2345.
- Fleuren MA, Paulussen TG, Dommelen P van, Buuren S van. Meetinstrument voor Determinanten van Innovaties (MIDI). ©TNO, 2012.
- Fleuren MAH, Paulussen TGWM, Van Dommelen P, Van Buuren S. Towards a measurement instrument for determinants of innovations. Int J Qual Heal Care. 2014;26(5):501–10.

***Appendix***

***Figure 1. Part of model of vulnerability during the childbirth continuum (Briscoe, et al., 2016)***


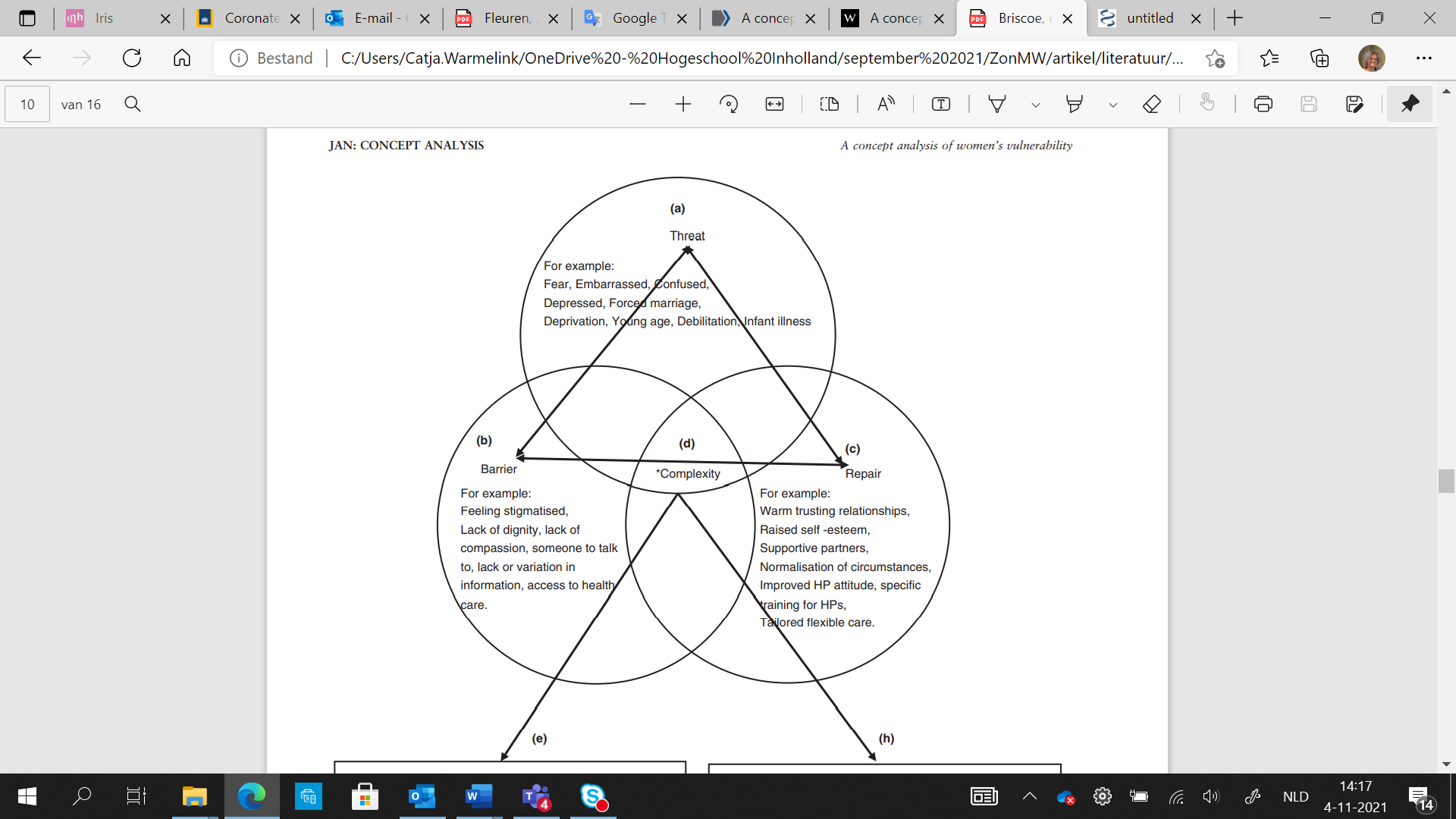


***Figure 2 Framework representing the implementation process and related categories of determinants***


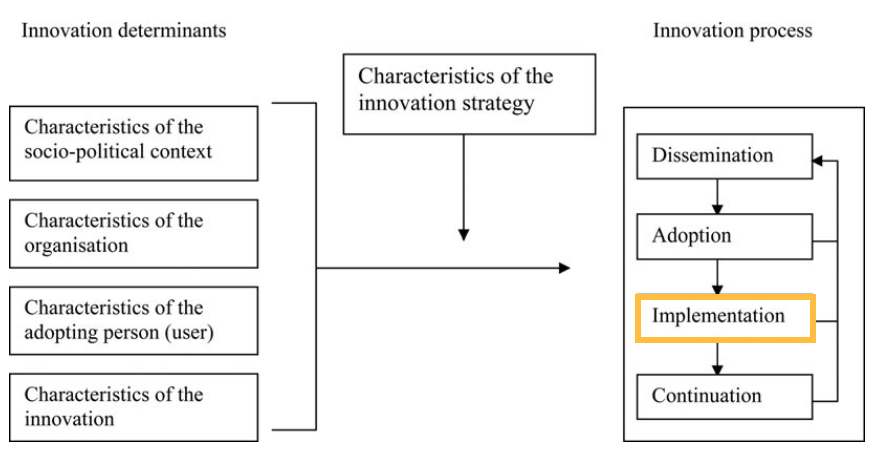


*During the interviews, the focus was on the ‘innovation’ determinants, in our case: the factors that influence the implementation of the intervention. The main focus was on the factors relating to the third phase in the innovation process, the implementation phase. During this phase, the intervention is incorporated into the daily routine* *(Fleuren, et al., 2014).*

***Figure 3 Measurement Instrument for Determinants of Innovations (MIDI): categories and determinants (Fleuren, et al., 2012)***

| Categories | Determinants |
| --- | --- |
| Determinants associated with the intervention | 1 Procedural clarity |
|  | 2 Correctness |
|  | 3 Completeness |
|  | 4 Complexity of intervention |
|  | 5 Compatibility |
|  | 6 Observability |
|  | 7 Relevance for women |
| Determinants associated with the health professional  (user) | 8 Personal benefits/drawbacks |
|  | 9 Outcome expectations |
|  | 10 Professional obligation |
|  | 11 Woman’s satisfaction |
|  | 12 Woman’s cooperation |
|  | 13 Social support |
|  | 14 Descriptive norm |
|  | 15 Subjective norm |
|  | 16 Self-efficacy |
|  | 17 Knowledge |
|  | 18 Awareness of content of intervention |
| Determinants associated with the organizational context of the professional | 19 Formal ratification by management |
|  | 20 Replacement when staff leave |
|  | 21 Staff capacity |
|  | 22 Financial resources |
|  | 23 Time available |
|  | 24 Material resources and facilities |
|  | 25 Coordinator |
|  | 26 Unsettled organization |
|  | 27 The information that is accessible about the use of the intervention |
|  | 28 Performance feedback |
| Determinants associated with social-political environment | 29 Laws and regulations |
