# Supplementary material for: Interventions for vulnerable pregnant women: Factors influencing culturally appropriate implementation according to health professionals: A qualitative study
Source: PLoS One. 2022 Aug 3;17(8):e0272249. doi: 10.1371/journal.pone.0272249 (PMC9348690; doi:10.1371/journal.pone.0272249)
Supplement: S3 File — (DOCX) [file pone.0272249.s003.docx]

**Supporting Table: Basic characteristics of participating health professionals**

|  | Inter-vention | Profession | Province | Work experience (in years)* | Experience with intervention (in years)* |
| --- | --- | --- | --- | --- | --- |
| 1 | CP | Hospital-based midwife | Groningen | 9 | 2 |
| 2 | CP | Primary care midwife | Drenthe | 21 | 2 |
| 3 | CP | Primary care midwife | Drenthe | 29 | 2 |
| 4 | CP | Primary care midwife | Groningen | 16 | <1 |
| 5 | CP | Primary care midwife | Overijssel | 8 | 2 |
| 6 | CP | Primary care midwife | Drenthe | 15 | 5 |
| 7 | other | 1. Primary care midwife 2. Resident ObGyn | Groningen  Groningen | 21  8 | 4  4 |
| 8 | CP | Primary care midwife | Groningen | 22 | 8 |
| 9 | NFP/SP | Primary care midwife | Drenthe | 29 | 5 |
| 10 | NFP/SP | GGD Youth nurse | Drenthe | 19 | 13 |
| 11 | NFP/SP | GGD Youth nurse | Friesland | 27 | 13 |
| 12 | NFP/SP | Home counselor Home care | Friesland | 35 | 12 |
| 13 | NFP/SP | Primary care midwife | Friesland | 5,5 | 5,5 |
| 14 | NFP/SP | 1. Manager 2. Manager | Friesland  Drenthe/Friesland | 25  9,5 | 13  3,5 |
| 15 | NFP/SP | GGD District nurse | Drenthe | 27 | 6 |
| 16 | CP | Primary care midwife | Friesland | 15 | 8 |
| 17 | other | Primary care midwife | Drenthe | 22 | n.a. |
| 18 | other | Coordinator MIM | Groningen | 13 | 13 |
| 19 | other | Primary care midwife | Friesland | 27 | 8 |
| 20 | other | a. Coordinator  b. Trainee social works | Friesland  Friesland | 0 | 7  n.a. |
| 21 | SCS | Primary care midwife | Friesland | 2 | 2 |
| 22 | SCS | Midwifery Student | Drenthe/Friesland | 0 | n.a. |
| 23 | SCS | Primary care midwife | Friesland | 24 | 1 |
| 24 | SCS | Primary care midwife | Groningen | 63 | 30 |
| 25 | SCS | Primary care midwife | Groningen | 46 | 6 |
| 26 | SCS | Primary care midwife | Groningen | 36 | 6 |
| 27 | SCS | Primary care midwife | Groningen | 32 | 1 |
| 28 | SCS | Hospital-based midwife | Friesland | 30 | 3,5 |
| 29 | SCS | Hospital-based midwife | Friesland | 42 | 12 |
| 30 | SCS | Hospital-based midwife | Friesland | 28 | 4,5 |
| 31 | SCS | Hospital-based midwife | Friesland | 32 | 5 |
| 32 | SCS | Hospital-based midwife | Friesland | 34 | 3 |
| 33 | SCS | Hospital-based midwife | Friesland | 30 | 6 |
| 34 | SCS | Hospital-based midwife | Friesland | 26 | 2,5 |
| 35 | SCS | Hospital-based midwife | Friesland | 40 | 11 |
| 36 | SCS | Hospital-based midwife | Friesland | 37 | 10 |

* *Approximately: a health professional may sometimes be temporarily out of work and / or an intervention may sometimes be temporarily suspended*

*CP = Centering Pregnancy: group consultations instead of individual prenatal consultations during pregnancy*

NFP/SP *=: extra support during and after pregnancy by a youth nurse*

*SCS= smoking cessation support (SCS)*

*n.a.= not applicable*
