# Supplementary material for: Interventions for vulnerable pregnant women: Factors influencing culturally appropriate implementation according to health professionals: A qualitative study
Source: PLoS One. 2022 Aug 3;17(8):e0272249. doi: 10.1371/journal.pone.0272249 (PMC9348690; doi:10.1371/journal.pone.0272249)
Supplement: Attachment — Submitted filename: Response to reviewers [PONE-D-21-05965].docx [file pone.0272249.s004.docx]

Groningen, the Netherlands, November 2021

Dear editor,

Thank you very much for considering our manuscript entitled “Interventions for vulnerable pregnant women: factors influencing culturally appropriate implementation according to health professionals: a qualitative study” for publication in your journal.

The manuscript has been carefully altered according to the helpful comments from you and the reviewers. A point-by-point reply to the reviewer is enclosed. The page numbers mentioned in our reactions refer to the 'Revised Manuscript with Track Changes'. We highlighted the changes within this document in yellow, because of that all changes can be tracked.

Hopefully you will be able to accept the revised manuscript for publication in PLOS ONE.

On behalf of all authors,

Yours sincerely,

Dr. Esther Feijen-de Jong

| **Number** | **Academic Editor** | |
| --- | --- | --- |
|  | **Comment** | **Response** |
| 1 | Please ensure that your manuscript meets PLOS ONE's style requirements, including those for file naming. | Thank you for this reminder, we hope that we have met the requirements you have set. |
| 2 | Please amend your current ethics statement to address the following concerns:  a) Did participants provide their written or verbal informed consent to participate in this study?  b) If consent was verbal, please explain i) why written consent was not obtained, ii) how you documented participant consent, and iii) whether the ethics committees/IRB approved this consent procedure | We highlighted our changes in yellow within the revised manuscript.  a) Participants provided written consent. We added the word *written* on page 11  b) NA |
| 3 | Please include a copy of the interview guide used in the study, in both the original language and English, as Supporting Information, or include a citation if it has been published previously. | A copy of the interview guide used in the study, in both the original language (Topic list Nederlands) and English (Topic list English), is included as Supporting Information |

| **Number** | **Comments to the author** | |
| --- | --- | --- |
|  | **Comment** | **Response** |
| 1 | Have the authors made all data underlying the findings in their manuscript fully available? | Unfortunately, we can not share the qualitative data sets (the interview transcripts) as additional supporting files in order to protect participants confidentiality. Although we did remove personal identifiers in the interview transcripts (such as the name and mail-address), the transcripts will likely to contain references to the contextual identifiers in individual stories and make individuals identifiable. On request and under certain conditions, we can provide access to the transcripts. These documents, however, are written in Dutch. |
| 2 | Is the manuscript presented in an intelligible fashion and written in standard English?  Reviewer #1: I would suggest reviewing the manuscript to improve grammar and clarity, possibly with the help of someone with full professional proficiency in English. | The manuscript is reviewed by the The University of Groningen Language Centre. Within the reviewing process, two (near-) native speakers have corrected the manuscript and translated the quotes. |

| **Number** | **Reviewer #1** | |
| --- | --- | --- |
|  | **Comment** | **Response** |
| 1 | Introduction a. The introduction could be strengthened by reviewing existing evidence regarding factors that influence implementation. The authors have, undoubtedly, reviewed this literature when devising their research plan and creating their interview guide.  b. I would also appreciate some evidence on the strength of the association between provider factors and the likelihood of implementation. If there is no such evidence, this should also be mentioned. | Thank you for this valuable comment.  a. Within the introduction we added evidence regarding factors that influence implementation (page 4):  Within this implementation process, many influencing factors *(i.e. factors related to the innovation (e.g. complexity or guidelines for using the innovation), the user (e.g.motivation, knowledge), the organization (e.g.resources), and the socio-political context (e.g.legislation))* can consequently interfere with a possible reducing effect of the intervention.  b. To our knowledge research examining the association between provider factors and the likelihood of implementation isn’t widely available. However, Vincenten et al. describe in their study that the acknowledgment of the key role of actors enhances the uptake of interventions.* We added a sentence within the introduction:  *In addition, especially the role of the professionals providing care might enhance the uptake of interventions.** It is important, therefore, to assess the implementation of interventions in actual care settings *from the perspectives of health care providers.*  Page 4  * Vincenten J, MacKay JM, Schröder-Bäck P, Schloemer T, Brand H. Factors influencing implementation of evidence-based interventions in public health systems – a model. Cent Eur J Public Health. 2019;27(3):198-203. doi: 10.21101/cejph.a5234. PubMed PMID: 31580554. |
| 2 | Study setting  This part is sound, and the authors make compelling arguments for choosing to focus on the Northern part of the Netherlands. It was important to note, as the authors did, that women in this area are mostly of Dutch origin. I think the authors should also provide some context for routine prenatal care in the Netherlands, as it is different than in many other high-income countries (e.g. midwives led, home birth is much likelier than in other high-income countries etc). | Thank you for this compliment.  The comment regarding the context for routine prenatal care is valuable. We added a text box on page 6 (“the maternity care system in the Netherlands”) explaining the Dutch maternity care system. |
| 3 | Selection of interventions  How many professionals were in the meeting in which a list of interventions was created? Was it a focus group, or one on one meetings with these professionals? | Within an advisory board meeting of our projects “Together we are strong”, involving 25 professionals (midwives, youth nurses, obstetricians, and community workers) we asked them (on 29-01-2019) to provide a list of the interventions they use most often.  To clarify this, we added the number *25 to the text (page 7)* |
| 4 | Box 1 – description of interventions  This is well-organized, and the interventions are clearly described. | Thank you |
| 5 | Research team and study participants  Thank you for providing context by describing each researcher’s background. You mention study participants have used/were using interventions for vulnerable pregnant women. Was this a recruitment criterion? If so, please provide reasoning for not including providers who did not use any of the interventions, who could shed light of why some practitioners refrain from implementing such interventions altogether. | Yes, we recruited only participants who used or were using interventions for vulnerable pregnant women, and having positive or negative experiences with the implementation process.  We agree with the reviewer, so we added in the discussion the following:  There *are* also limitation*s* that needs to be considered when interpreting the results. ../..  *Many interventions have been developed for vulnerable pregnant women, but some professionals are not familiar with these interventions or do not adopt them. In future research the professionals who do not use interventions can be interviewed to shed light on why some professionals refrain from implementing interventions.*  Page 32 |
| 6 | Data collection  This section is concise and well-written.  a. As a follow-up to my comment to the previous section, please provide information on inclusion and exclusion criteria, and also on the number of individuals who contacted the research team but then refused or dropped out of the research, in accordance with the COREQ checklist (this can be added to the results section).  b.Were some individuals screened out by the research team, based on their characteristics? | a. We added: *Half of the professionals approached did not respond to the request or indicated that they did not wanted to participate.*  Page 10  b. To achieve variation in our sample, final recruitment was targeted at professionals in various categories of adoption of the intervention: innovators, majority, and laggards (Paulussen & Bessems, 2017)  No professionals were screened out by the research team, based on other characteristics.  We added: *To achieve variation in our sample, final recruitment was targeted at professionals in various categories of adoption of the intervention.* (Page 9) |
| 7 | Data analysis  This section is clear and well-written. | Thank you. |
| 8 | Results  Table 1 is well-constructed and clear. Please provide clarification on how “awareness of context of intervention” differs from “familiarity with intervention”. | To be able to learn more about the content of the intervention (determinant ‘Awareness of content of intervention’), one first needs to know that it exists (awareness). To be clearer, we have changed the description of the determinant ‘Familiarity with intervention’ in: E*xtent to which the user knows about the existence of the intervention.* (Page 16) |
| 9 | Theme 1: Attitude of the health professional  Lines 257-267: interviewees 36 and 3 are quoted several times each; was this theme represented broadly among participants? | Yes, it was broadly represented among the participants. This is reflected in the variety of quotes. In this specific part (attitude of the health professional) we quoted participants 8, 26, 17, 19, 36, 3, 29 and 6.  However, to make it more clearly, we have removed and added the following quotes:  We removed the following quotes: *Whatever I say, it won’t change” (#3, CP).*  *“Your baby will soon be here, just have another cigarette” (#3, CP). “A group of people who are not open to conversations” (#36, SCS).*  And added the following quote:  *People who don't want it, you can talk to them as much as you want but it's not going to happen. So that, that's what I don't think is worth investing my time and energy in. (# 29, SCS)*  Page 279 |
| 10 | Theme 2:MIDI determinants  a. One overarching comment: this section is quite long and could use some editing and re-organization. I suggest summarizing data pertaining to MIDI determinants previously described in a shortened format, and perhaps organize in a table. Lengthier descriptions should be, in my opinion, reserved for the new determinants described by the authors. Another option is to focus solely on the new determinants, provide more detailed descriptions, and mention that previously described determinants have been validated in this study group.    b. Please provide additional details on the two last determinants: the social environment and culture; and local/central government. Particularly, how do these differ from other determinants (e.g. the examples for government pertain to funding; how does that determinant differ from the funding determinant?).  c. I’d also be curious to know if interviewees were asked to choose the most salient determinants, or if the researchers noted the most commonly cited determinants, in order to offer some clarity on the relative importance of different determinants. | a. Thank you for this comment. We also struggled with this part of the results in the process of writing this paper. From the interviews, we collected a very wide range of data. Within our team we had a long discussion about this issue and decided to present information specifically for vulnerable pregnant women on each determinant of the MIDI in order to inform readers about each aspect of the influencing factors of intervention implementation. (page 19 of the manuscript) Validation of the MIDI was not a goal, yet adding locally grounded information from professionals in the field was our goal. Therefore, we used the MIDI as a template and described every determinant in order to be able to provide a well-structured analysis of the data we have collected. We hope the reviewer can agree with our reflections on this.  b. Within the Netherlands the local municipality decides whether to fund interventions. For instance, when a woman moves during her pregnancy, she may not be able to complete a program she is participating in in her new municipality. This is an example of funding related to the local/central government. Next to this, also organisations can decide if they want to fund an intervention or not. For instance, midwifery practices can decide, due to a lack of money or time, not to offer group prenatal care (Centering Pregnancy).  The reviewer is right, because we put two quotes in the wrong place it is not clear enough. We have therefore moved these two quotes to “Determinants associated with the sociopolitical environment”.  The following quotes were moved:  *“That subsidy, of course, can give the municipality a push to get it started” (#10, NFP/SP).*  *“That whole pot of money, of course. It’s a big problem here as well. The municipality is perfectly aware that there’s no money but the population that needs it is growing” (#11, NFP/SP).*  Page 27  c. Interviewees were not asked to choose the most salient determinants. The interviewers were instructed to ask open-ended questions. They were, however, known to the MIDI determinants which were included in the topic list as background information. Thus, we did not ask for an ordering in level of importance. |
| 11 | Discussion  Please provide an explanation to the reason only one determinant (attitude of the health professional) was described separately. | The reviewer raises an important point here. The reason for describing the attitude of the HCP as a separate theme is because we found this to be such an important issue that has implications not only for the process of implementation but for the overall care process. The other new determinants identified have specific focus on the implementation process and are therefore included as determinants.  We added the following on page 14:  *The reason for describing the attitude of the HCP as a separate theme is because we found this to be such an important issue that has implications not only for the process of implementation but for the overall care process.* |
| 12 | Attitude of the health professional  a. The term “stigma” is mentioned in the results when describing this determinant, and should also be included in the discussion. I would advise the authors to broaden the discussion of stigma in healthcare, particularly in prenatal care.  b. Also include here the context of the study as interviewees cared mostly for native Dutch population (rather than for immigrants).  c. Consider also including a review of germane literature pertaining to culturally-competent care. Please provide justification as to why this is an important issue.  d.What do we know of the impact of culturally competent vs. stigmatized care on health outcomes? | a. Thank you, we agree that stigma is a very important topic to address. Within the discussion we used the words as stereotype and homogenization, which in our opinion can lead to stigma. However, to be more clear we added the following:  *Which can lead to stigmatization of a group of persons. (page)*  *AND*  *A negative attitude or stigma towards vulnerable pregnant women (in this study, mostly native Dutch women living in poverty) can have a considerable influence on women’s healthcare-seeking behaviour or may lead to not offering interventions to women at all, resulting in a lower quality of care.*  Page 28  b. We included a sentence regarding the context of the study:  *(in this study, mostly native Dutch women living in poverty)*  Page 28  c. We acknowledge this comment of the reviewer.  Within our conclusions we state: “We highlight the need to challenge stereotypical views and attitudes in order to provide relation-centred care since this is extremely important to provide culturally appropriate care that leads to better outcomes for vulnerable women and their babies.” Within this conclusion we use the concept of relationship-centred care (RCC), which recognizes that the nature and quality of relationships in health care influence the process and outcomes of health care. RCC is founded on four principles:  1. Personhood matters  2. Affect and emotion are important  3. Relationships do not occur in isolation  4. Maintaining genuine relationships is necessary for health and recovery, and is morally valuable.* (Soklaridis et al, 2016)  If we relate this concept to culturally-competent care we came to the conclusion that cultural competent care is part of the concept of relationship-centred care and therefore we have not described this concept as a separate issue.  d. We recognize that interactions between health care providers and patients/clients influence the course and outcomes of care.* Therefore we highlight the importance of providing relationship-centred care.  We added some text and three references to this sentence in our manuscript:  A negative attitude towards vulnerable pregnant women (in this study, mostly native Dutch women living in poverty) can have a considerable influence on women’s healthcare-seeking behaviour, *engagement of care, adherence to treatment,* or may result in women not being offered interventions at all, resulting in a lower quality of prenatal care which is *associated with poorer health outcomes*. (e.g. low birth weight, preterm birth)  Page 28  **Soklaridis S, Ravitz P, Adler Nevo G, Lieff S. Relationship-centred care in health: A 20-year scoping review. Patient Experience Journal. 2016; 3(1):130-145. doi: 10.35680/2372-0247.1111.*  **Stangl, Anne L et al. “The Health Stigma and Discrimination Framework: a global, crosscutting framework to inform research, intervention development, and policy on health-related stigmas.” BMC medicine vol. 17,1 31. 15 Feb. 2019, doi:10.1186/s12916-019-1271-3*  **Heaman MI, Martens PJ, Brownell MD, Chartier MJ, Derksen SA, Helewa ME. The Association of Inadequate and Intensive Prenatal Care With Maternal, Fetal, and Infant Outcomes: A Population-Based Study in Manitoba, Canada. J Obstet Gynaecol Can. 2019 Jul;41(7):947-959. doi: 10.1016/j.jogc.2018.09.006. Epub 2019 Jan 11. PMID: 30639165.* |
| 13 | Strengths and limitations  a.Please change the title to strengths and limitation*s*  b. Using the MIDI may have also “led” interviewees to speak of the experience using the MIDI conceptualization.  c. Another limitation worth considering is that by selecting the interventions most often used, the authors introduce a bias which should be mentioned in the limitations section (some interventions may not be studied, since they failed to become popular, and factors influencing these interventions will not be studied).  d. The study has also included health providers caring mostly for native Dutch population. This is both and advantage of the study (indicated that stigma is an important determinant when caring for native, disadvantaged population), and a disadvantage, as different themes may emerge when interviewing providers caring for more ethnically diverse population. | a.We changed the title in *Strengths and limitations*  Page 31  b. We don’t think that could have happened because the interviewees did not know about the MIDI. We only used the MIDI as background information, and as a template to analyse our data.  c. We agree with the reviewer. We added: *In future research, professionals who do not use the interventions selected in this study or use other interventions may be interviewed to shed light on the influencing factors of such interventions.*  Page 32  d. That’s indeed possible. However, it was our aim to describe the perspectives of healthcare providers from the northern part of the Netherlands. These providers care mostly for the Dutch native population. We certainly think the reviewer has a point so we added in the limitations section the following:  *Furthermore, different themes may emerge when interviewing providers caring for more ethnically diverse populations.*  Page 31 |
| 14 | Recommendations In the last paragraph, you write “above all, attention needs to be paid to the attitude of health professionals…”. This statement requires justification. Is this the determinant found to be most important by the authors? Also, consider collapsing it with the previous paragraph, pertaining to training of health professionals. | We agree that it needs better justification. Indeed, we think that this overarching theme is very important and therefore have repeated it here. However, the reviewer's comment to merge this with the training of health professionals is actually a better option. Therefore, we have moved this text to the section providing recommendations for health professionals. Following the reviewer's suggestion, we have merged the last two paragraphs of the recommendations section (page 33):  *In education, it is extremely important to socialize students so that they become health professionals who open up (implicitly and explicitly) to every vulnerable group that they encounter in their daily practice. Practice also needs to pay attention to this issue. Possibly practice and education can join hands to develop a plan together. Throughout the educational curriculum, attention should be paid to challenging stereotypical views and creating positive views of and values towards vulnerable groups. Also, attention needs to be paid in educational programmes and in practice to understanding interventions, the need for them and the complexity of implementing them in a culturally appropriate manner. Using a framework may help to organize and structure the many influencing determinants. A social map that includes all the interventions for vulnerable pregnant women also needs to be developed.* |

| **Number** | **Reviewer #2** |  |  |
| --- | --- | --- | --- |
|  | **Comment** | | **Response** |
| 1 | While the statistical coding and analysis was done carefully and systematically, participants were recruited through a snowball system. This sampling raises questions about the extent to which respondents are representative of all professional groups working in the field under study (only one obstetrician, and is a mother (lay person?) from the 'mother Inform mothers' concept in the sample?) Although many results may be generalizable, a sample stratified by occupational groups and length of time working in the field might reveal more specific results. | | In this qualitative study it was not our goal that the respondents were representatives of all occupational groups, as is common in quantitative studies. We have tried to improve the validity and verifiability of our research by techniques as triangulation of interviewers and analists, peer debriefing, member checking and negative case analysis.  To be more transparent we added: The study participants were *primary care and hospital-based midwives, gynaecologists, youth and district nurses, regional managers of maternity care assistants*, who are using or have used interventions for vulnerable pregnant women in the North of the Netherlands (in the provinces of Groningen, Friesland and Drenthe).  Page 9  We added: *To achieve variation in our sample, final recruitment was targeted at professionals in various categories of adoption of the intervention.*  Page 9  We added a table of background characteristics of the participants as Supporting Information. |
| 2 | a. For example, ‘bonding’ with clients is described as helpful in improving health behavior. However, it does fit less to the role of the doctor (obstetrician) than to the ‘mother to mother’- concept, which seems more promising to reach pregnant women through the peer effect. Therefore, the experiences of health workers (such as midwifes, doctors and nurses) in building relationshops with women, may differ from the findings of more peer-orientated staff (including ‘mothers inform mothers’). The difference should be presented and discussed as it may require adapted improvements strategies.  b. In addition, the used intervention tools vary from specific interventions for vulnerable pregnant women to general, e.g. specific smoking cessation. Do these differences not affect the respective goals and outcomes, which would then have to be evaluated diffently? | | a. We understand what the reviewer points out. However, our goal was not to present differences between the health professionals. Within future research the point of the reviewer can shed light to these issues.  b. That could potentially be a problem. However, we conducted research at an overarching level to present generic elements around the factors that influence implementation of interventions for vulnerable groups. In the interviews, we focused on these group, even when we discussed a more general intervention. |
| 3 | Another issue is the length of employment in this area of work, as both experience an the potential for frustration increase over time. This should also be taken into account when presenting the results. Is there a trend to be observed here? This information could be supportive to implement the research results into improved practice. | | This was not the aim of this paper, we do agree that length of employment and experience can have the potential for frustration increase. However, from the data of our interviews we can not identify a trend. This should be examined in quantitative research. We are currently conducting a cross-sectional survey study. It is possible that from that survey we can answer this important question. |
| 4 | Furthermore, the semi-structured questionnaire and a demographic table with information about gender, professional groups, and length of work experience should be added to display a clear picture of participants. | | We added a copy of the interview guide used in the study, in both the original language (Topiclist Nederlands) and English (Topiclist English). (Supporting Information).  We added a table of background characteristics of the participants as Supporting Information. |
