# Supplementary material for: Interventions for vulnerable pregnant women: Factors influencing culturally appropriate implementation according to health professionals: A qualitative study
Source: PLoS One. 2022 Aug 3;17(8):e0272249. doi: 10.1371/journal.pone.0272249 (PMC9348690; doi:10.1371/journal.pone.0272249)
Supplement: Attachment — Submitted filename: Review for manuscript PONE R1.docx [file pone.0272249.s005.docx]

Review for manuscript PONE-D-21-05965-R1

Thank you for sending the revised manuscript. I believe the revisions have greatly strengthened the manuscript and have few minor comments to make.

Methods

Study Setting

Thank you for adding a text box contextualizing the maternity care system in the Netherlands. I believe it significantly adds to the understanding of international readers. Please add a reference to the text box in the text of this section.

Selection of interventions

Thank you for clarifying the setting in which interventions were selected. I would add to the manuscript text all details you provided in your response to my comment (i.e., not only the number of participants, but also that this was a part of an advisory board meeting for a project) and also an explanation of the nature of the *Together we are strong* project. This sounds like a very appropriate setting in which to collect the list of interventions – I just want all details to be clear for readers (e.g. if they want to replicate a similar intervention selection process in a different country).

Research team and study participants

Thank you for adding information both to the methods section and to the discussion on study participants being limited to those who use the interventions. I would even be more explicit in the discussion (e.g. “**our study only recruited participants who used interventions**, the perspective of practitioners who do not use any intervention should be assessed etc”), as some people will not read the methods section carefully.

Data collection

Thank you for the modifications. Please add the absolute number of practitioners approached (was it 80? 81? 79?). Please also add information on how the categories of adoption of intervention were assessed – this is super interesting and a unique strength of your study. Please also mention in the text no individuals were screened out by the study team.

Theme 2: MIDI determinants

a. The explanation for keeping all MIDI determinants in is sound and reasonable. Please add a short version of this explanation to the manuscript. Please clearly separate between existing MIDI determinants and new determinants described by the authors.

Discussion

Strengths and limitations

c. In my comment, I referred to interventions that failed to become popular, and were therefore not mentioned. This is a different limitations than the one related to the study participants. I would also add this to the limitations (your analysis is limited to interventions that were quite successful to begin with, and does not shed light of why some interventions gain no traction among providers).

* Thank you for including your interview guide and characteristics of study participants as supporting information. I suggest to include the characteristics of study participants in the manuscript (if table number limits allow it), and in any case, to include a reference to these two important sources in the manuscript text.
