# Supplementary material for: Interventions for vulnerable pregnant women: Factors influencing culturally appropriate implementation according to health professionals: A qualitative study
Source: PLoS One. 2022 Aug 3;17(8):e0272249. doi: 10.1371/journal.pone.0272249 (PMC9348690; doi:10.1371/journal.pone.0272249)
Supplement: Attachment — Submitted filename: Response to reviewer [ PONE-D-21-05965-R1].docx [file pone.0272249.s006.docx]

**Response to reviewer**

| **Number** | **Reviewer** | |
| --- | --- | --- |
|  | **Comment** | **Response** |
| 1 | Methods  Study Setting  Thank you for adding a text box contextualizing the maternity care system in the Netherlands. I believe it significantly adds to the understanding of international readers. Please add a reference to the text box in the text of this section. | We added a reference to the textbox, namely:  De Geus, M., & Cadée, F. (2012). Midwifery in the Netherlands. *Utrecht: Royal Dutch Organisation of Midwives*.  *Page 6* |
| 2 | Selection of interventions  Thank you for clarifying the setting in which interventions were selected. I would add to the manuscript text all details you provided in your response to my comment (i.e., not only the number of participants, but also that this was a part of an advisory board meeting for a project) and also an explanation of the nature of the *Together we are strong* project. This sounds like a very appropriate setting in which to collect the list of interventions – I just want all details to be clear for readers (e.g. if they want to replicate a similar intervention selection process in a different country). | Thank you for this constructive feedback. The reviewer is right that we can be more clear about the setting in which the interventions were selected. Therefore, we removed and added:  Removed:  First, at a meeting, we asked 25 maternity care professionals (midwives, youth nurses, obstetricians and community workers) to provide a list of the interventions that they use most often.  Added:  *During an advisory board meeting (29 January 2019) of the 'together we are strong' projects, attending professionals were asked to provide a list of the interventions they used most often. The 'together we are strong' projects are two projects with the aim to improve the implementation of interventions for vulnerable pregnant women in the northern part of the Netherlands. Both projects share an advisory board that meets at least twice a year. This advisory board involves 25 professionals (midwives, youth nurses, obstetricians, and community nurses).*  *Page 7* |
|  | Research team and study participants  Thank you for adding information both to the methods section and to the discussion on study participants being limited to those who use the interventions.  I would even be more explicit in the discussion (e.g. “**our study only recruited participants who used interventions**, the perspective of practitioners who do not use any intervention should be assessed etc”), as some people will not read the methods section carefully. | Thank you for this comment, we made it more clear by adding in the discussion section:  *Furthermore, our analysis is limited to interventions that are quite successful, as a result we do not provide insight into why some interventions are not finding traction with providers (44). Moreover, in our study, we only recruited health care providers who used interventions. Future research should assess the perspectives of professionals who do not use interventions designed for vulnerable pregnant women to shed light on the reasons for this non-use.*  *Page 33* |
|  | Data collection  Thank you for the modifications. Please add the absolute number of practitioners approached (was it 80? 81? 79?).  Please also add information on how the categories of adoption of intervention were assessed – this is super interesting and a unique strength of your study.  Please also mention in the text no individuals were screened out by the study team. | We changed this into:  *We approached around 80 potential participants. Half of the professionals approached did not respond to the request or indicated that they did not wanted to participate. We aimed to include 40 participants in order to keep the scope of the research topic broad.*  Page 10  To make it more clear we removed: To achieve variation in our sample, final recruitment was targeted at professionals in various categories of adoption of the intervention. (Page 10)  We added: *To achieve variation in our sample, final recruitment was targeted at professionals in various categories of adoption of the intervention: innovators, majority, and laggards (Paulussen & Bessems, 2017). Certain groups can be distinguished based on how quickly they adopt an intervention (Paulussen & Bessems, 2017). We deliberately looked for professionals who were "innovators" and "early adaptors. These change agents were the first to pick up new interventions, are well-known leading figures who are respected in the profession and therefore of great importance for dissemination to large groups, the 'majority'. 'Laggards' are afraid of innovation and often need requirements/guidelines from the profession for the final push to adoption. During the iterative process of recruitment and data analysis, we checked whether the voice of the 'majority' and 'laggards' was heard. We also spoke (as a negative case study) with someone who was able to successfully offer an intervention in the Western part of the Netherlands, but failed to do so in the North of the Netherlands.*  Page 10  *Other characteristics of healthcare professionals were not taken into account during the recruitment process.*  Page 10 |
|  | Theme 2: MIDI determinants  The explanation for keeping all MIDI determinants in is sound and reasonable. Please add a short version of this explanation to the manuscript.  Please clearly separate between existing MIDI determinants and new determinants described by the authors. | *At page 20 we have written:*  *In the interviews, we found many determinants that apply to the implementation process in general. However, due to the large amount of data that we generated, we will only present the determinants that are specifically related to the factors that influence implementation for vulnerable pregnant women.*  *Furthermore, to make it more clear we added the word specifically at page 13:*  *Table 1 shows the factors that specifically influence the implementation of interventions for pregnant women in vulnerable situations from the perspective of health professionals.*  We have highlighted the new determinants in orange  In the text we added:  *In addition, six new determinants were inductively constructed by the researchers (shown highlighted in orange in Table 1).*  Page 13 |
|  | Discussion  Strengths and limitations  c. In my comment, I referred to interventions that failed to become popular, and were therefore not mentioned. This is a different limitations than the one related to the study participants. I would also add this to the limitations (your analysis is limited to interventions that were quite successful to begin with, and does not shed light of why some interventions gain no traction among providers). | We agree with the reviewer and added:  *Furthermore, our analysis is limited to interventions that are quite successful, as a result we do not provide insight into why some interventions are not finding traction with providers (44).*  Page 32 |
|  | * Thank you for including your interview guide and characteristics of study participants as supporting information. I suggest to include the characteristics of study participants in the manuscript (if table number limits allow it),  and in any case, to include a reference to these two important sources in the manuscript text. | We added in the text:  See supplementary File S1 and S2 for the topic list in Dutch and in English.  Page 11  AND  See Supplementary File S3 for the basic characteristics of participating health professionals  Page 13 |
