# Supplementary material for: Interventions for vulnerable pregnant women: Factors influencing culturally appropriate implementation according to health professionals: A qualitative study
Source: PLoS One. 2022 Aug 3;17(8):e0272249. doi: 10.1371/journal.pone.0272249 (PMC9348690; doi:10.1371/journal.pone.0272249)
Supplement: Attachment — Submitted filename: Response to reviewer [ PONE-D-21-05965-R1].docx [file pone.0272249.s007.docx]

**Response to the reviewer**

| **Number** | **Reviewer** | |
| --- | --- | --- |
|  | **Comment** | **Response** |
| 1 | Reviewer #2: The authors did not address my comment on the first resubmission. The manuscript states that only professional health care workers were included. The concept of "mothers for mothers" reads like a lay approach, so please clarify, especially for the international readership, whether or to what extent this group is trained and/or paid for their work in addition to their personal experience as a mother. | Thank you for this comment. Actually we interviewed the coordinator of the mothers for mothers intervention. This person is a paid, highly educated coordinator. To make that more clear we added within the methods section (page 8):    Box 1 (Mothers inform mothers) *‘A paid, highly educated coordinator is responsible for recruiting, selecting, inducting and supervising volunteers as well as for matching volunteers with (expectant) mothers’*    In addition we added in tabel S3 (#18) coordinator *‘MIM’* |
